# Fermented *Lactobacillus plantarum* GKD7 prevents osteoarthritis pain and progression in a preclinical* in vivo* model

**DOI:** 10.7150/ijms.129270

**Published:** 2026-03-30

**Authors:** Chin-Jung Hsu, Kun-Tsan Lee, Li-Chai Chen, You-Shan Tsai, Yen-Po Chen, Yen-Lien Chen, Chin-Chu Chen, Yen-You Lin, Tzu-Ching Chang, Chih-Hsin Tang

**Affiliations:** 1 School of Chinese Medicine, China Medical University, Taichung, Taiwan.; 2 Department of Orthopedic Surgery, China Medical University Hospital, Taichung, Taiwan.; 3 Department of Post-Baccalaureate Medicine, National Chung-Hsing University, Taichung, Taiwan.; 4 Department of Orthopedics, Taichung Veterans General Hospital, Taichung, Taiwan.; 5 Department of Pharmacy, Tajen University, Pingtung, Taiwan.; 6 Biotech Research Institute, Grape King Bio Ltd., Taoyuan City, Taiwan.; 7 Institute of Food Science and Technology, National Taiwan University, Taipei City, Taiwan.; 8 Translational Medicine Center, Shin Kong Wu Ho-Su Memorial Hospital, Taipei, Taiwan.; 9 Department of Pharmacology, School of Medicine, China Medical University, Taichung, Taiwan.; 10 Chinese Medicine Research Center, China Medical University, Taichung, Taiwan.; 11 Department of Medical Laboratory Science and Biotechnology, College of Medical and Health Science, Asia University, Taichung, Taiwan.

**Keywords:** Osteoarthritis, Fermented GKD7, Probiotics

## Abstract

Osteoarthritis (OA) brought on by aging damages joints and impairs function. Probiotics are known to be safe to eat, and many of them have positive bioactivity for human health issues. *Lactobacillus plantarum* GKD7 mitigates inflammatory cytokine production and has potential for OA treatment. This study investigated whether fermented GKD7 is effective at preventing the advancement of OA. Anterior cruciate ligament transaction-related bone discomfort and OA are lessened by fermented GKD7. Fermented GKD7 prevented the breakdown of aggrecan and type II collagen alpha 1 chain (COL2A1) by lowering pro-inflammatory cytokines interleukin-1β (IL-1β) and tumor necrosis factor-α (TNF-α), as well as the chondrolytic factors metalloproteinase-3 (MMP)-3, MMP-13, and a disintegrin and metalloproteinase with thrombospondin motifs 5 (ADAMTS5). This activity prevented cartilage deterioration and bone loss. Our results show that fermented GKD7 improves the prevention of OA formation.

## Introduction

One of the most common degenerative illnesses, osteoarthritis (OA), has become one of the most common health issues as a result of advances in medicine and longer life expectancies. Over 528 million people worldwide suffered with OA, and over a ten-year period, its prevalence rose by 114.5%, according to the Global Burden of Disease research 1, 2. Subchondral bone sclerosis, cartilage degradation, and inflammation of the synovial area are pathological features of OA. At the time of diagnosis, these traits often result in irreparable joint stiffness and discomfort 3, 4. The only available therapies for OA aim to lessen discomfort or stop the disease's progression; there is presently no recognized cure.

Chronic inflammation of the synovial tissues is closely related to structural damage, joint pain, and the release of synovial fluid, all of which are critical in increasing inflammation and tissue degradation in OA 5, 6. The severity of knee OA is highly correlated with proinflammatory cytokines like tumor necrosis factor-α (TNF-α) and interleukin-1β (IL-1β), as well as synovium-related factors like metalloproteinase-3 (MMP)-3, MMP-13, and a disintegrin and metalloproteinase with thrombospondin motifs 5 (ADAMTS5), which may also be linked to the disease's progression 7-10. Increased amounts of inflammatory mediators and degradative agents cause the cartilage matrix's constituents, including collagen II and aggrecan, to degrade 11, 12. According to pertinent research, OA may be treated with inflammation-reduction techniques 5, 13.

Probiotics have been prominent targets for study and development in the treatment of OA since they are considered safe for eating and many exhibit therapeutic bioactivity for human illnesses. *Lactobacillus plantarum* is a thoroughly researched probiotic bacterium, with demonstrated effects of decreasing inflammation in human hosts, among various other benefits 14. As a result, *Lactobacillus plantarum* has been utilized in research involving various human diseases in both clinical and pre-clinical trials, including osteoporosis, arthritis, abdominal pain and bowel disease 15-17. In our earlier work, we documented that *Lactobacillus plantarum* GKD7 mitigates the progression of OA by inhibiting inflammatory cytokine production associated with it 18. Both live and dead GKD7 exhibit anti-inflammatory functions 19. This study investigated whether fermented GKD7 is effective at preventing the advancement of OA. In this study, we discovered that fermented GKD7 prevent the onset of OA induced by anterior cruciate ligament transection (ACLT)* in vivo*.

## Materials and Methods

### Preparation of fermented GKD7

GKD7, which was isolated from Taiwanese pickles, was grown in de Man-Rogosa-Sharpe medium for lactobacilli (Merck, Germany). The fermented GKD7 was made in accordance with our earlier report 20.

### ACLT animal model

The National Laboratory Animal Center in Taipei, Taiwan, provided us with eight-week-old male Sprague Dawley (SD) rats that weighed between 300 and 350 g. They were randomly assigned to three groups: sham surgery (controls), ACLT alone, and ACLT with fermented GKD7 (100 mg/kg). The ACLT surgeries were carried out using the procedure described in our earlier reports 21, 22.

According to our prior guidelines, the weight-bearing incapacitance test was used every week to assess spontaneous discomfort following ACLT based on variations in dynamic weight bearing between the resting right and left hind limbs 23, 24. Separate sensor plates were used to quantify the difference in dynamic weight bearing between the left and right hind limbs throughout a 10-second period. To determine the average score, three consecutive measurements were made for each animal on each test day. The data are displayed as a percentage of body weight to take animal differences in body weight into consideration.

### μ-CT measurements

After receiving medication for six weeks, the rats were put to death. Their intact right knee joints were scanned using a SkyScan 2211 μ-CT scanner (Bruker; Kontich, Belgium) and processed using CTAn software, in accordance with our earlier methods 23, 25. In short, the femurs and tibias were fixed in 4% formaldehyde and then 70% ethanol after the skin and muscle tissue were removed. To avoid beam-hardening artifacts, the scans were carried out across a 180° rotation at a voltage of 70 kVp, a current of 290 µA, and a 0.5 mm aluminum filter. 59 reoriented slices (0.5 mm each) were chosen for additional investigation after image reconstruction. In the subchondral trabecular bone region of the medial tibial plateau, manually created regions of interest (ROIs) with an uneven contour.

### Histological analysis

As previously reported, histological changes in OA tissue were examined under an optical microscope using hematoxylin and eosin (H&E) and safranin-O/fast green stains 26, 27. Knee joint tissues were preserved in 4% formaldehyde and then decalcified using 10% EDTA. The next step was ethanol dehydration. The specimens were cut into 5 µm thick sections for histological staining after being embedded in paraffin blocks. The Osteoarthritis Research Society International (OARSI) histological assessment system was used to evaluate the structural changes in the cartilage of the central weight-bearing region of the medial tibial plateau 28. The severity of OA and the depth of lesions are displayed by this system using staging and grading scores, respectively.

### Immunohistochemistry (IHC) staining

The immunohistochemistry analysis was conducted using the Leica Novolink Polymer Detection system (Leica Biosystems Inc., IL, USA), as detailed in reference 29, 30. Tissue slices were treated with 3% BSA following a quick application of 3% hydrogen peroxide. The slices were stained with diaminobenzidine substrate after primary antibodies were applied, and then they were treated for an hour with a secondary antibody combined with peroxidase. Both the percentage of immunoreactive positive cells and the strength of the immunoreactive signals were quantitatively scored, with a range of 0 to 7. Immunoreactive signal intensity was rated on a scale of 1 to 5 (from weak to strong). The following score was applied to the percentage of immunoreactive cells: 0 represents no signal, 1 represents less than 10% positive cells, 2 represents 10-29% positive cells, 3 represents 30-59% positive cells, and 4 represents 60-100% positive cells. To reduce observer bias, two impartial assessors who were blind to the treatment groups performed the scoring analysis.

### Statistical analysis

GraphPad Prism 5.0 was used to perform statistical analyses for quantified data. The mean ± standard deviation (S.D.) is used to display the data. Results from two groups and more than two groups were compared using the paired sample t-test and one-way ANOVA followed by Bonferroni post hoc testing, respectively. In every instance, a *p*-value of less than 0.05 indicated statistical significance.

## Results

### Fermented GKD7 do not affect the body weight growth curve

**Figure 1:**
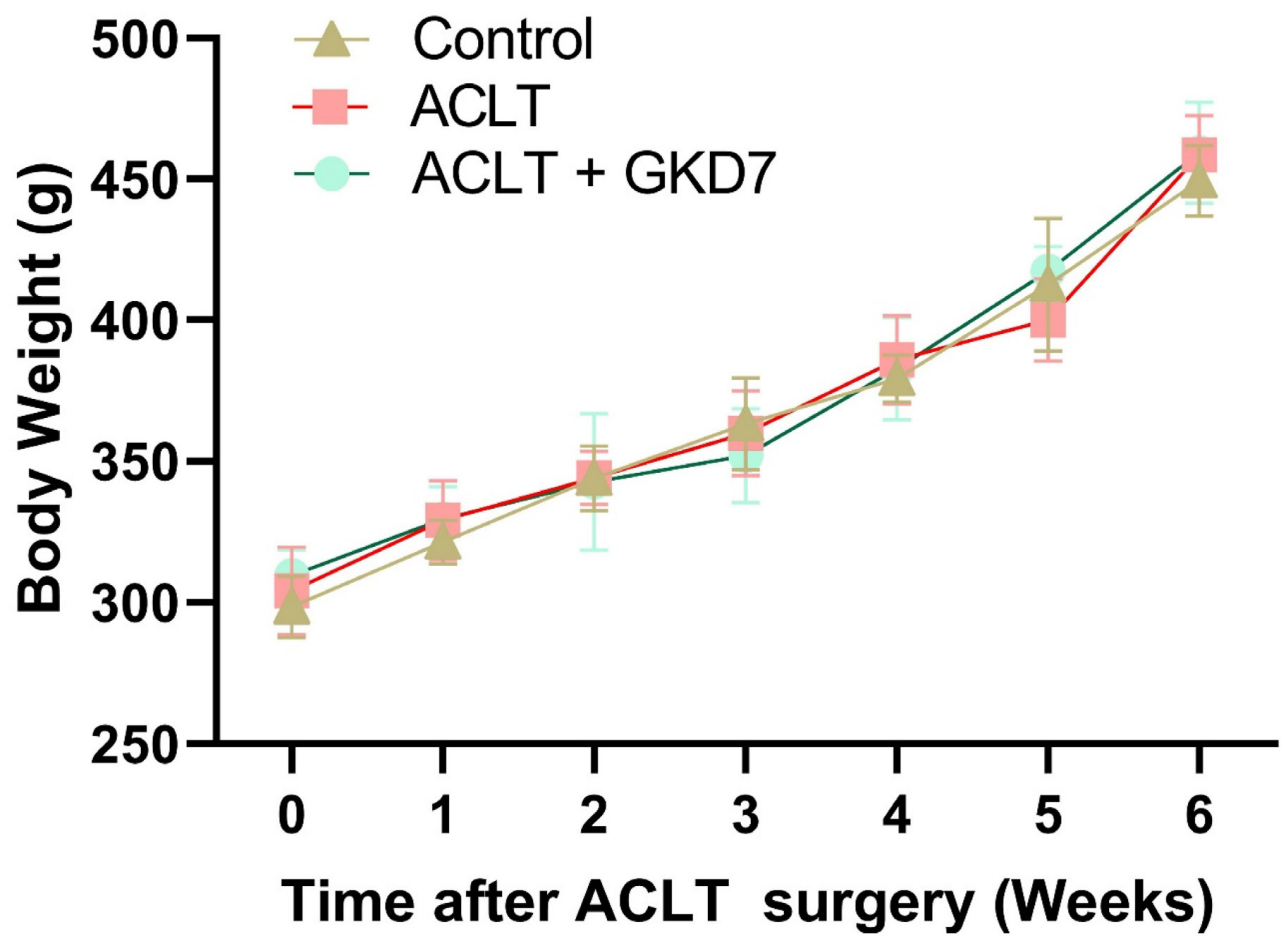
** Increase in body weight throughout the experimental phase.** Throughout the course of the experiment, body weight was measured.

We used a rat model of ACLT-induced knee arthritis to investigate the preventative advantages of fermented GKD7. Assessments of pain behavior and histology examinations were carried out to investigate the underlying causes. The day prior to surgery, the rats' body weights were measured, and this process was repeated each week until the animals were killed. Throughout the experiment, the body weight of each group climbed gradually, and there were no appreciable variations between the groups (Fig. 1). Our findings indicate that fermented GKD7 has no detrimental impact on body weight.

### Fermented GKD7 ameliorates OA pain

**Figure 2:**
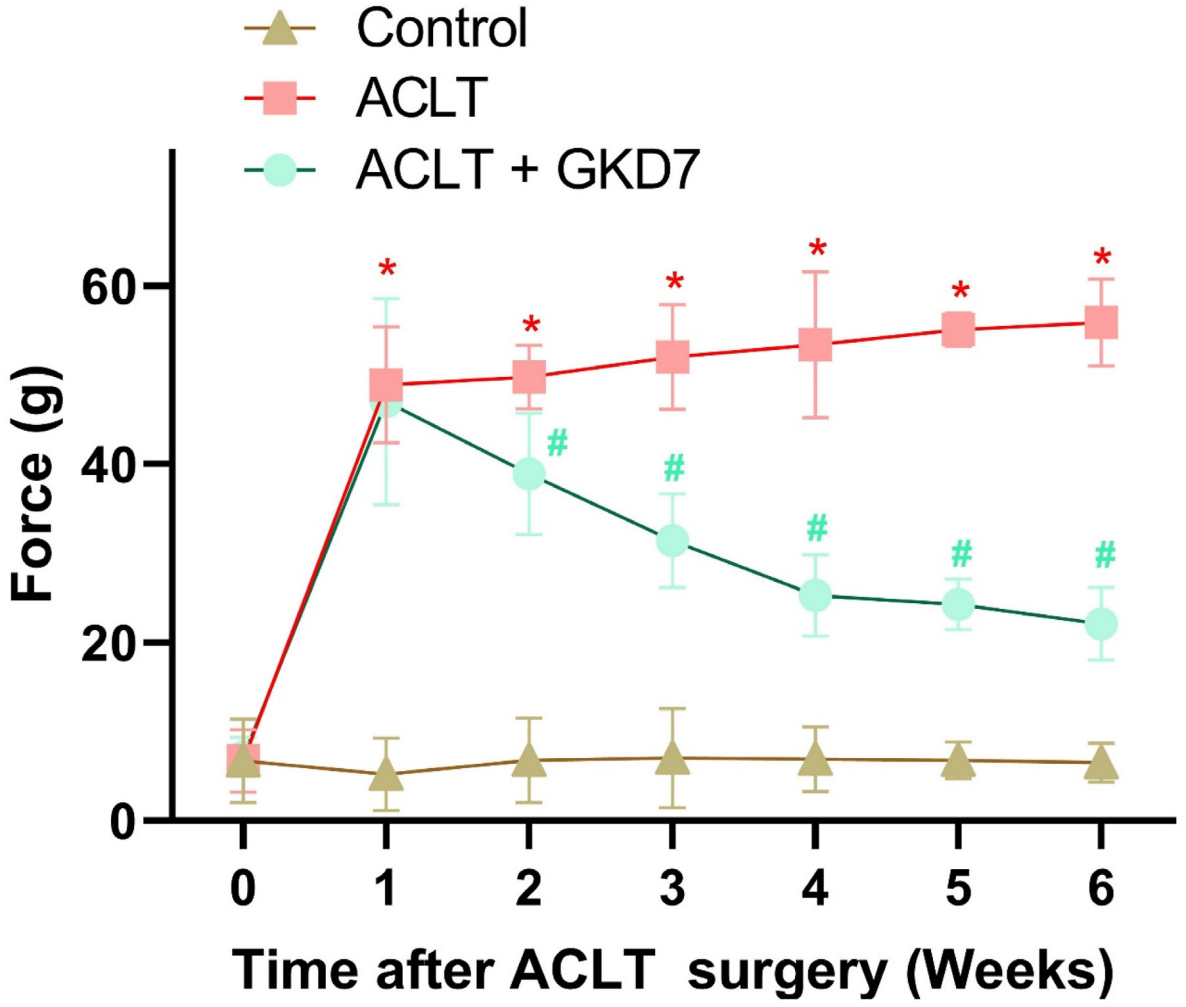
** Fermented GKD7 decelerates ACLT-induced bone pain.** Every week, weight-bearing behavioral testing was conducted to assess deficits in weight-bearing forces. * *p*<0.05 compared with the control group; # *p*<0.05 compared with the ACLT-only group.

The static weight-bearing incapacitance test was used to assess the rats' pain behavior. During the first week after surgery, all groups displayed a markedly different weight-bearing posture (Fig. 2). This notable imbalance grew more noticeable in the ACLT animals during the course of the investigation. Nonetheless, the ACLT plus fermented GKD7 group showed significant improvements in pain-related behavior (Fig. 2). These results suggest that fermented GKD7 effectively lessens OA-related discomfort.

### Fermented GKD7 protects against ACLT-induced osseous and cartilage damage in an ACLT-induced OA model

**Figure 3:**
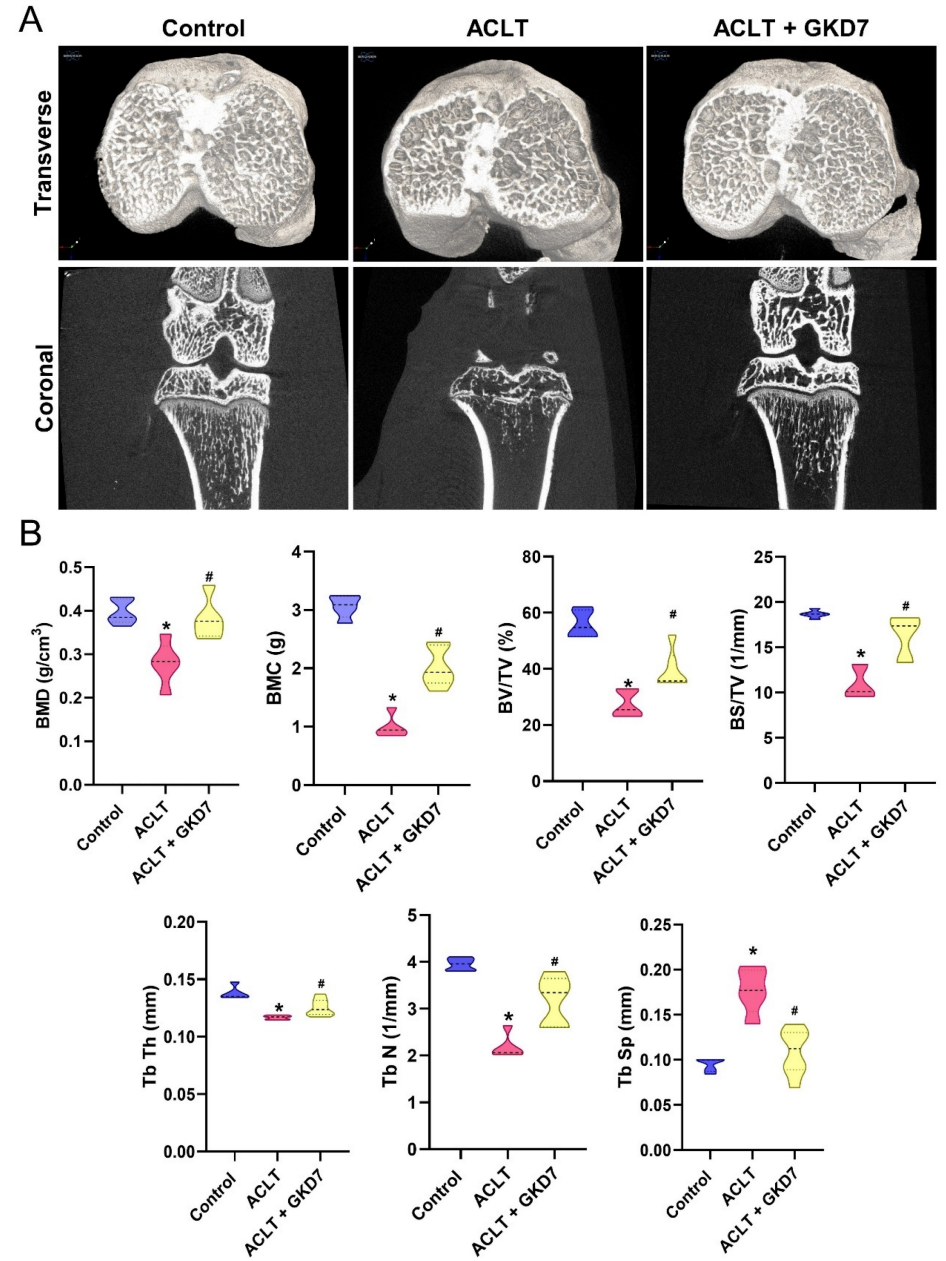
** Fermented GKD7 ameliorates osseous damage in the ACLT-induced OA knee joint.** (A) Representative micro-CT images from knee subchondral bone. (B) Quantitative analyses of BMD, BMC, BV/TV, BS/TV, Tb.Th, Tb.N, and Tb.Sp. * *p*<0.05 compared with the control group; # *p*<0.05 compared with the ACLT-only group.

Six weeks after ACLT surgery, μ-CT was used to measure changes in trabecular microarchitecture. The OA lesion caused by ACLT surgery was demonstrated by the significant bone degradation observed in ACLT mice compared to controls (Fig. 3). Quantitative evaluation revealed a decrease in trabecular thickness (Tb.Th), trabecular number (Tb.N), trabecular separation (Tb.Sp), bone mineral density (BMD), bone mineral content (BMC), bone volume/tissue volume ratio (BV/TV), and bone surface to tissue volume ratio (BS/TV) in ACLT rats (Fig. 3). In addition, compared to the ACLT group, rats treated with fermented GKD7 exhibited notable improvements in bone microstructure (Fig. 3).

**Figure 4:**
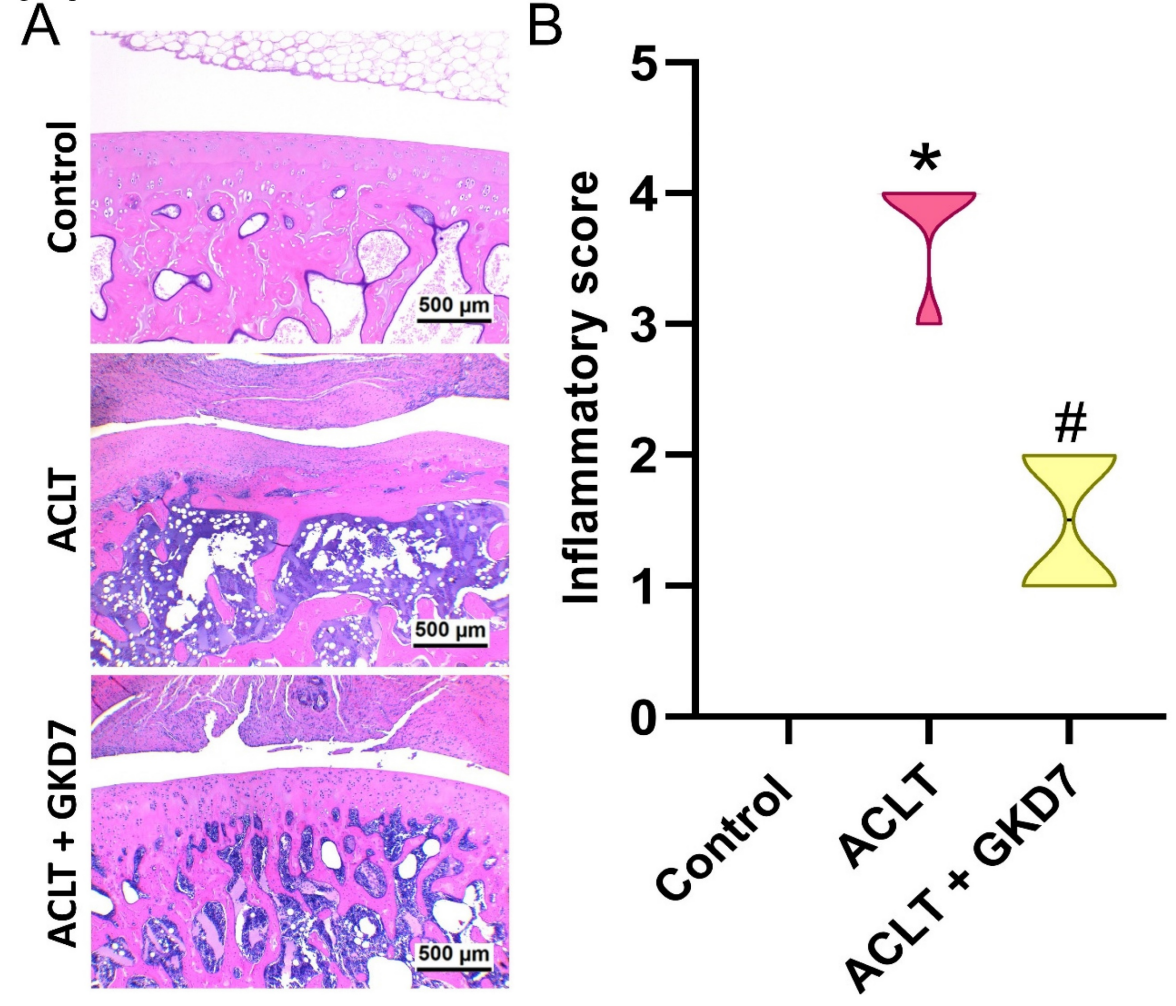
** Fermented GKD7 blocks ACLT-induced synovial inflammation and cartilage degradation.** (A) Histological sections from knees stained with H&E. (B) Quantitative analyses of synovium scores. * *p*<0.05 compared with the control group; # *p*<0.05 compared with the ACLT-only group.

**Figure 5:**
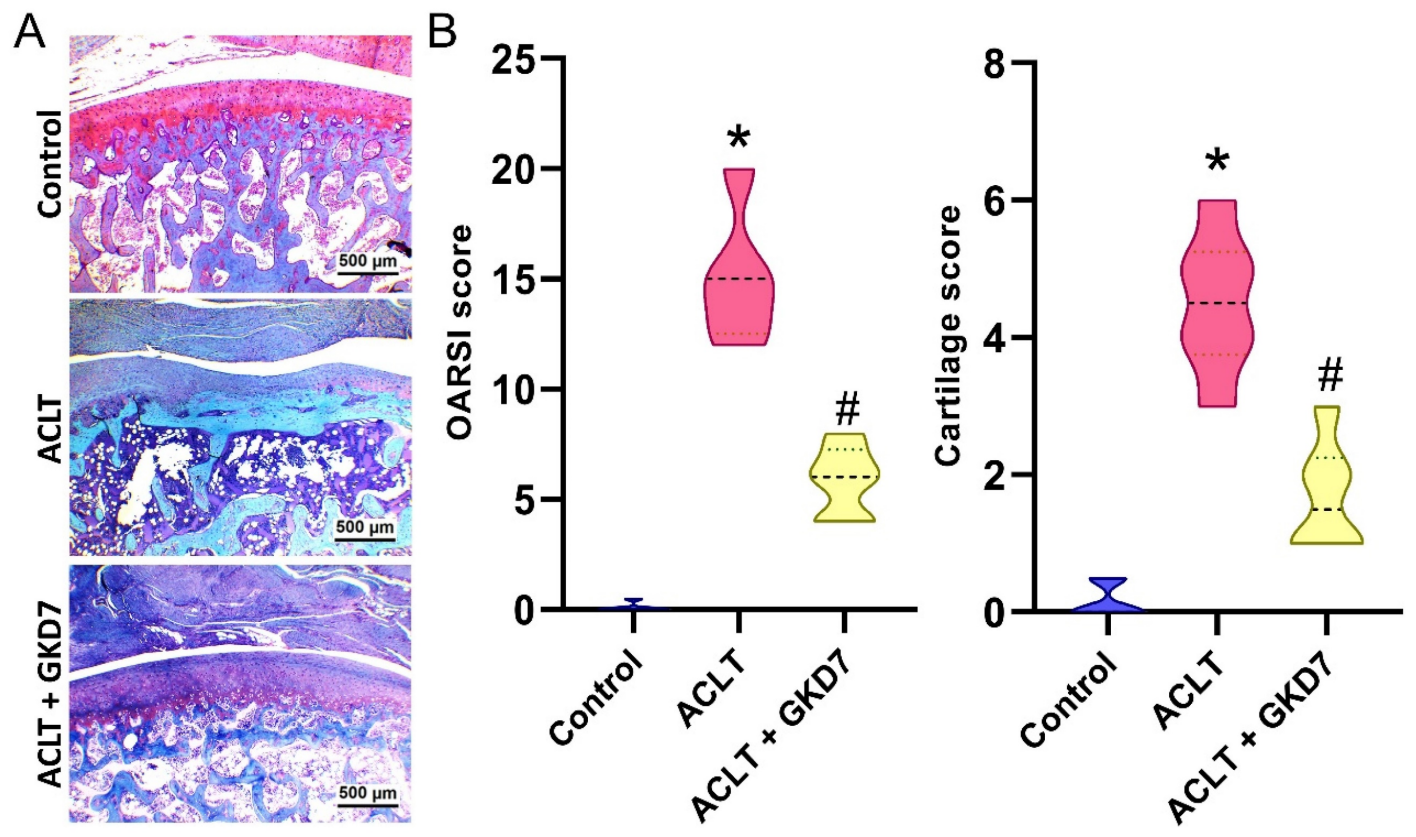
** Fermented GKD7 blocks ACLT-induced cartilage breakdown.** (A) Histological sections from knees stained with Safranin-O. (B) Quantitative analyses of OARSI and cartilage scores. * *p*<0.05 compared with the control group; # *p*<0.05 compared with the ACLT-only group.

Histological analysis of the ACLT knee groups using H&E and Safranin-O/Fast Green staining showed hyperplasia of the synovial lining and articular cartilage degradation (Fig. 4&5). The quantification of inflammation, OARSI scores, and cartilage scores showed that the ACLT plus fermented GKD7 group had less pathological alterations in cartilage tissue and less synovial tissue hyperplasia than the ACLT group (Fig. 4&5).

### Fermented GKD7 suppress proinflammatory cytokine production and cartilage degradation

**Figure 6:**
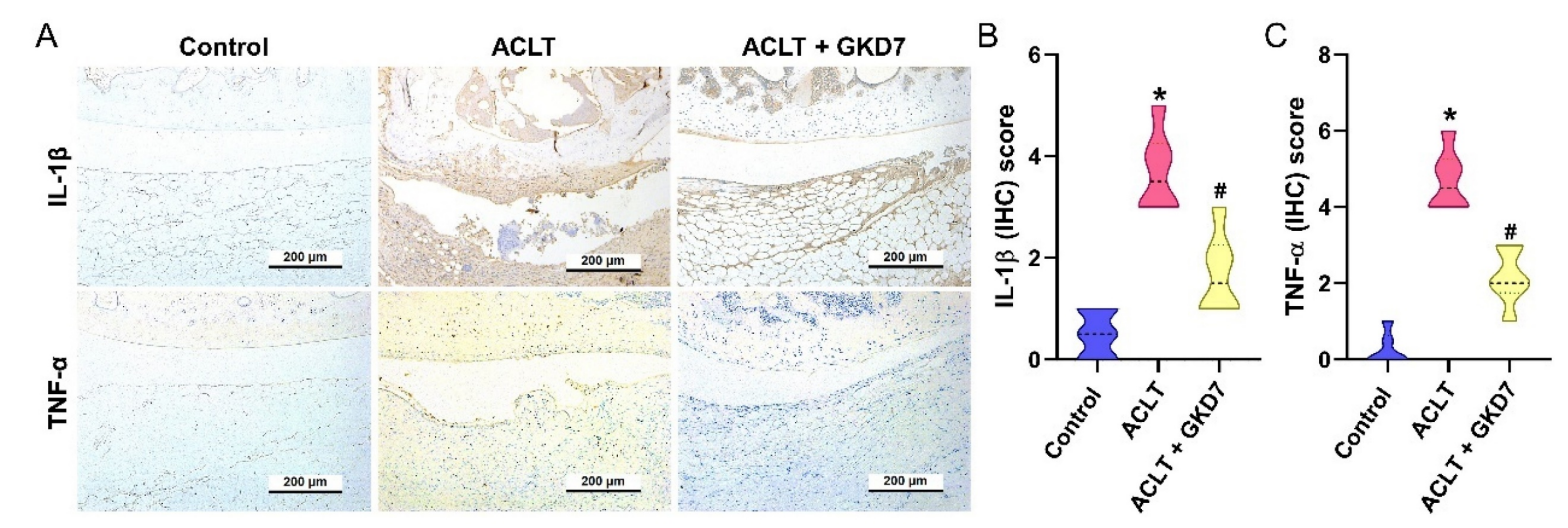
** Fermented GKD7 diminishes the induction of IL-1β and TNF-α in ACLT-induced OA articular cartilage.** Immuno-histochemistry analysis and scoring of IL-1β (A, B) and TNF-α (A, C) in rat knee joint cartilage. * *p*<0.05 compared with the control group; # *p*<0.05 compared with the ACLT-only group.

**Figure 7:**
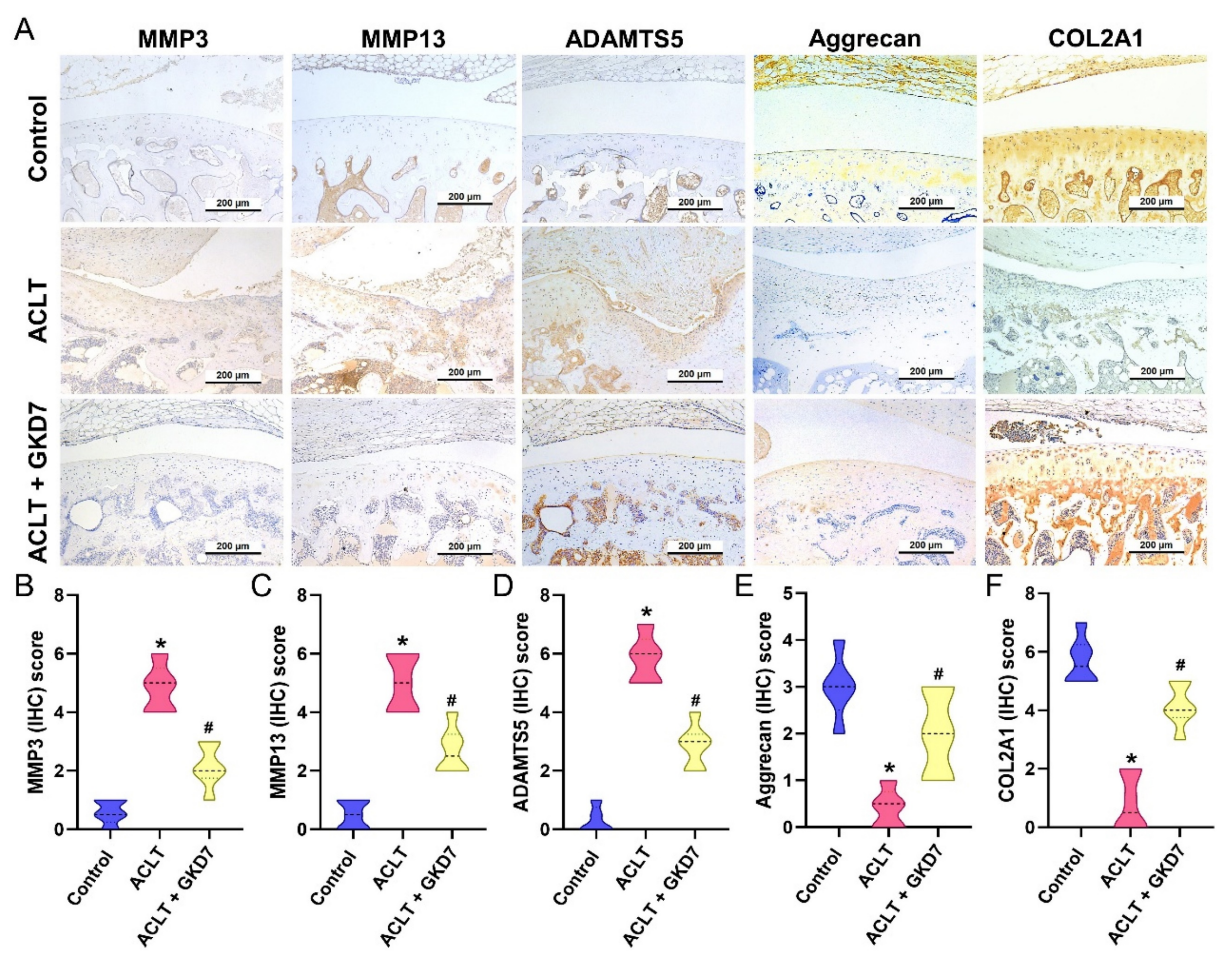
** Fermented GKD7 reserves the expression of aggrecan and COL2A1 accompanying with suppression of MMP-3, MMP-13 and ADAMTS5 in ACLT-induced OA articular cartilage.** (A) Immuno-histochemistry analysis MMP3, MMP-13, ADAMTS5, aggrecan and COL2A1 in rat knee joint cartilage. (B-F) Scoring of the immunosignals of MMP3, MMP-13, ADAMTS5, aggrecan and COL2A1. * *p*<0.05 compared with the control group; # *p*<0.05 compared with the ACLT-only group.

The ACLT group's synovial tissue produced significantly more TNF-α and IL-1β, according to the IHC study, suggesting a rise in inflammatory activity. As seen in Figure 6, this increase was significantly lower in the ACLT plus fermented GKD7 group. IHC labeling of MMP-3, MMP-13, ADAMTS5, aggrecan, and type II collagen alpha1 chain (COL2A1), the building block of articular cartilage, was used to further assess cartilage metabolism. Compared to the ACLT group, the ACLT plus fermented GKD7 group had reduced levels of MMP-3, MMP-13, and ADAMTS5 and greater levels of aggrecan and COL2A1 (Fig. 7).

## Discussion

Numerous probiotic strains used as supplements have shown promise in treating human conditions such as gastrointestinal, respiratory, and arthritic problems 31. Probiotics may help people with OA-related pain by improving gut microbiota and lowering inflammation through several OA therapy pathways, according to preclinical and clinical research 32. Probiotics may also prevent cartilage degradation and the development of OA models, according to preclinical research 33. For instance, the use of a combination of the probiotic strains *Lactiplantibacillus plantarum* and *Lacticaseibacillus paracasei* notably reduced cartilage damage at the medial femoral condyle in a mouse model with medical meniscus destabilization 33. Additionally, daily treatment with *Streptococcus thermophilus* alleviated OA pain and cartilage damage in pre-clinical OA model 34. We previously found that live and dead GKD7 exhibits anti-inflammatory and chondroprotective functions in an OA model 19. Here, our investigation further revealed that fermented GKD7 reduces OA-related pain and progression in an ACLT-promoted OA model. Fermented GKD7 inhibits inflammatory cytokine expression and cartilage degradation *in vivo*. Fermented GKD7 also serves as a potential supplement for OA management.

Synovial inflammation, cartilage degradation, and pain behavior are all impacted by OA, a chronic inflammatory illness 35, 36. Inflammatory cytokines, including TNF-α and IL-1β, contribute significantly to the development of OA by producing anomalies in chondrocyte metabolism, enhanced inflammatory responses, and joint discomfort 13, 37. Furthermore, OA patients have significantly greater levels of TNF-α and IL-1β in their serum and synovial tissue, according to clinical findings from earlier studies 21, 38. During pre-clinical studies, IL-1β and TNF-α are crucial targets for developing effective OA therapy strategies. Our ACLT-induced OA model showed that ACLT surgery mimics clinical characteristics, leading to elevated production of TNF-α and IL-1β in cartilage and synovial tissue. The administration of fermented GKD7 definitely led to a downregulation of TNF-α and IL-1β production in cartilage and synovial tissues, indicating that the anti-OA characteristics of fermented GKD7 are derived from its capacity to inhibit TNF-α and IL-1β synthesis.

The gel-like matrix of cartilage is mostly composed of collagen and the proteoglycan aggrecan. While COL2, the primary matrix component, creates a fibrous network foundation, proteoglycans attract water parts to produce a gel that maintains the cartilage's strong and inflated qualities 39. The cartilage matrix helps chondrocytes maintain stability and a regulated metabolism 40. Diseases associated with cartilage, such as joint inflammation and articular deterioration, develop when chondrocytes are unable to maintain metabolic homeostasis within the cartilage matrix 41, 42. Here, we found that ACLT inhibited the expression of aggrecan and COL2A1 while enhancing the synthesis of the chondrolytic proteins MMP-3, MMP-13, and ADAMTS5. Fermented GKD7 restores chondroprotective properties and delays the progression of OA by decreasing the expression of chondrolytic proteins.

Although the preclinical results with fermented GKD7 show encouraging chondroprotective effects in the ACLT rat model, there are a number of significant obstacles in applying these findings to actual OA patients. The majority of the evidence supporting probiotics in OA is currently found in animal models; human clinical trials are still few, small-scale, and frequently reveal inconsistent structural disease change rather than variable or mild effects on pain and inflammation. Significant obstacles include strain-specific efficacy, ideal dosage and formulation, inter-individual variation in gut microbiota composition impacting colonization and systemic anti-inflammatory responses, and the requirement for longer-term studies to validate mechanisms such as gut-joint axis modulation in humans. Conducting well-powered, randomized, double-blind, placebo-controlled clinical trials in patients with early to moderate-stage OA to assess pain alleviation and functional outcomes could be one way to strengthen the conclusion in the future.
